# African wild dog movements show contrasting responses to long and short term risk of encountering lions: analysis using dynamic Brownian bridge movement models

**DOI:** 10.1186/s40462-022-00316-7

**Published:** 2022-03-31

**Authors:** Ben Goodheart, Scott Creel, Milan A. Vinks, Kambwiri Banda, Johnathan Reyes de Merkle, Anna Kusler, Chase Dart, Kachama Banda, Matthew S. Becker, Peter Indala, Chuma Simukonda, Adrian Kaluka

**Affiliations:** 1 Department of Ecology, Montana State University, 310 Lewis Hall, Bozeman, MT 59717 USA; 2 Zambian Carnivore Programme, PO Box 80, Mfuwe, Eastern Province Zambia; 3 Insitutioned För Vilt, Fisk Och Miljö, Sveriges lantbruksuniversitet, Umeå, Sweden; 4 Montana Fish Wildlife and Parks, 490 North Meridian Road, Kalispell, MT 59901 USA; 5 Zambia Department of National Parks and Wildlife, Private Bag 80, Lusaka, Zambia

**Keywords:** Competition, Brownian bridge movement model, Prey depletion, African wild dog, Lion, Kafue National Park

## Abstract

**Background:**

Prey depletion is a threat to the world’s large carnivores, and is likely to affect subordinate competitors within the large carnivore guild disproportionately. African lions limit African wild dog populations through interference competition and intraguild predation. When lion density is reduced as a result of prey depletion, wild dogs are not competitively released, and their population density remains low. Research examining distributions has demonstrated spatial avoidance of lions by wild dogs, but the effects of lions on patterns of movement have not been tested. Movement is one of the most energetically costly activities for many species and is particularly costly for cursorial hunters like wild dogs. Therefore, testing how top-down, bottom-up, and anthropogenic variables affect movement patterns can provide insight into mechanisms that limit wild dogs (and other subordinate competitors) in resource-depleted ecosystems.

**Methods:**

We measured movement rates using the motion variance from dynamic Brownian Bridge Movement Models (dBBMMs) fit to data from GPS-collared wild dogs, then used a generalized linear model to test for effects on movement of predation risk from lions, predictors of prey density, and anthropogenic and seasonal variables.

**Results:**

Wild dogs proactively reduced movement in areas with high lion density, but reactively increased movement when lions were immediately nearby. Predictors of prey density had consistently weaker effects on movement than lions did, but movements were reduced in the wet season and when dependent offspring were present.

**Conclusion:**

Wild dogs alter their patterns of movement in response to lions in ways that are likely to have important energetic consequences. Our results support the recent suggestion that competitive limitation of wild dogs by lions remains strong in ecosystems where lion and wild dog densities are both low as a result of anthropogenic prey depletion. Our results reinforce an emerging pattern that movements often show contrasting responses to long-term and short-term variation in predation risk.

**Supplementary Information:**

The online version contains supplementary material available at 10.1186/s40462-022-00316-7.

## Background

The ecology of large carnivores makes them inherently rare (Colinvaux 1979), and they are experiencing population declines and range reduction due to habitat loss, direct persecution, and prey depletion [15, 21, 56]. In addition to these problems, interspecific competition strongly structures many large carnivore guilds. Because subordinate competitors are limited by dominant competitors, conservation efforts are further complicated for these species [9, 23, 29, 43, 53]. Prey depletion is an emerging threat that affects many carnivore populations in developing countries [74], in a manner that is likely to interact with the effect of interspecific competition [13]. The population density of dominant competitors such as lions (*Panthera leo*) is strongly correlated with prey density, and decreases in response to prey depletion [67, 69]. Densities of subordinate competitors such as wild dogs (*Lycaon pictus*) and cheetah (*Acinonyx jubatus*) are not tightly correlated with prey density, but are negatively correlated with the density of their dominant competitors [11, 30, 36, 47, 48, 63]. Recent research has shown that the reduction of dominant competitors (lions) does not necessarily release subordinate competitors (wild dogs), if the low density of dominant competitors is caused by prey depletion [28]. Much research has described the effects of dominant competitors on the distribution and abundance of subordinate carnivores in ecosystems with intact prey communities [11, 43, 57], but we know little about these effects when both prey and dominant competitors are reduced (a condition that is increasingly common).

Large carnivores often compete by interference. Wild dogs are strongly affected by kleptoparasitism by spotted hyenas [11, 21, 22, 29] and intraguild predation by lions [11, 30, 47]. According to Gause’s law, selection should favor adaptations that reduce niche overlap between pairs of competing species (particularly in subordinate competitors), and for large carnivores these adaptations usually reduce overlap in the set of prey species that is hunted, temporal patterns of hunting activity, or space use. The effects of competition on the times and places that subordinate competitors hunt have been studied extensively [11, 19, 20, 47, 63], but there has been almost no research on how their movement patterns respond to the risk of encountering dominant competitors.

Understanding the effects of competition on movement is important because movement is one of the most energetically costly behaviors for many species [1, 64]. These costs are particularly important for cursorial hunters like the African wild dog [17, 29]. Using allometric relationships that estimate the costs of transport from Taylor et al. [64], Creel et al. [17] estimated that African wild dogs expend 3.04 MJ per hour of movement. Using doubly labelled water, Gorman et al. [29] found a very similar value, 3.14 MJ per hour of movement. Using data on the number of kills made per day, the consumable mass of each kill and the digestible energy content of consumed tissue, Creel & Creel [11] found that wild dogs in the Selous Game Reserve obtained 2.5 kg of food/individual/day and 5.8 MJ/kilogram. Taking the mean (3.07 MJ/ h) of the two published estimates of the cost of transport, an African wild dog would require 0.53 kg of food to offset the cost of one extra hour of movement, which represents a 21.2% increase relative to their daily intake. Using data on the limits of sustained metabolic activity Gorman et al. [29] also see [35, 61] suggested that wild dogs could not sustain a decrease in energy intake of this magnitude due to loss of kills from kleptoparasitism by hyenas (and lions). By the same logic, an increase in energy expenditure of this magnitude to avoid predation, would also be unsustainable. This line of reasoning suggests that understanding the effects of competition on the movements of wild dogs (and other subordinate competitors) may help us to understand how competition restricts their distribution and abundance [26, 40].

Most species are affected by interspecific competition, and anthropogenic effects can alter competitive interactions. Understanding these effects is critical for the conservation and management of endangered subordinate carnivores [13]. Wild dogs are an excellent species with which to study these issues, because they are limited by interference competition with lions and spotted hyenas and are always found at lower densities than their dominant competitors [11]. Anthropogenic prey depletion has recently been identified as an important driver of low wild dog population density [28]. Problematically, low-density wild dog populations often reach a local extinction threshold after relatively small-scale disturbances, exemplified by wild dog populations in the Ngorongoro crater, the Serengeti plains, and Liuwa plains [11, 19, 27].

The Kafue National Park (KNP), which forms the backbone of the Greater Kafue Ecosystem (GKE), has long been considered a stronghold for wild dogs in Zambia and neighboring nations that encompass the greater Kavango-Zambezi Transfrontier Conservation Area (KAZA) [17]. However, the density of ungulate prey in KNP has been severely reduced by decades of poaching pressure stemming from the illegal bushmeat trade [51, 70]. While the dynamics are not fully understood, the bushmeat trade in savanna Africa is driven by national and international demand from urban and rural areas, and lack of employment and economic opportunity in communities adjacent to protected areas [42]. The largest taxa within the wild ungulate guild are disproportionately targeted due to their greater economic value, which has led to greater reductions in larger ungulate species than medium and small species [41]. The loss of larger ungulates in Kafue has led to prey-base homogenization, niche compression, and increased dietary overlap within the large carnivore guild [13], and contributed to low densities of both lions and wild dogs [28, 69]. Survival rates for wild dogs in KNP are comparable to those in stable, high-density populations, suggesting that low wild dog density is primarily driven by prey depletion, rather than direct additive mortality from wire snares or other human impacts (which would yield lower survival rates) [28]. Wild dog packs in Kafue are smaller than most other ecosystems, and home-ranges in Kafue are the largest recorded for the species, suggesting that the combination of low prey and competitor densities could have strong effects on movement, but the effects of prey availability and dominant competitors on wild dog movements have not been studied in Kafue or elsewhere.

On one hand, low lion density might reduce the need for movements that serve to avoid risky situations. On the other hand, low prey density could strengthen competition, which might keep the effect of dominant competitors on movement strong. Here, we tested how wild dog movements were affected by lions, prey, and other variables in an ecosystem with anthropogenically reduced densities of prey and dominant competitors. Specifically, we estimated the Brownian motion variance derived from dynamic Brownian Bridge Movement Models (dBBMMs) fit to several thousand locations from GPS collared wild dogs in several packs over several years, to obtain a measure of spatial displacement accounting for both the speed and linearity of movement. We then tested for effects on Brownian motion variance of long-term space-use by lions, short-term proximity to lions, environmental predictors of prey density, and local anthropogenic effects, to reveal what processes most strongly affected wild dog movements.

## Methods

### Study area

Our study was conducted in the central and northern portions of the Kafue National Park and the surrounding Mumbwa-West, Kasonso-Busanga, and Lunga-Busanga Game Management Areas (GMAs). The Greater Kafue Ecosystem (GKE) is located in western Zambia (S14.5394, E26.0782), and totals 66,000 km^2^, comprised of the Kafue National Park and surrounding GMAs, which are managed for hunting, wildlife protection, farming, and fishing. The GKE forms the northernmost portion (and 13%) of the Kavango-Zambezi Transfrontier Conservation Area (KAZA TFCA), which spans Angola, Botswana, Namibia, Zambia, and Zimbabwe. The ecosystem is dominated by miombo woodland (*Brachystegia* and *Julbernadia spp*.) and a mosaic of Acacia woodland, termitaria woodland, riverine woodland, savannah grassland, and seasonally inundated grasslands. The region receives an average of 1,020 mm of total rainfall per year, with a rainy season between December and April with extensive flooding, and a dry season between May and November [22].

### Data collection

We deployed satellite GPS collars (Model TGW 4270: Telonics Inc., Mesa, Arizona, USA) on at least one individual in 10 wild dog packs from 2017 to 2020. Because wild dog packs almost invariably move as a highly cohesive unit, we analyzed data from one individual per pack (to balance sampling among packs). Wild dog locations were recorded twice daily, once in the morning between 08:00 & 08:30 and once in the late evening between 18:00 & 19:00 for a total of 9,624 unique locations. These times are at the ends of crepuscular peaks of movement by wild dogs, so that consecutive locations typically included one complete morning or evening movement period [10]. This sampling regime does not examine fine-scaled patterns within each movement period. Rather, it samples complete hunting periods, from the initiation of movement until the next rest period. We also deployed satellite collars (Model TGW-4570, Telonics Inc., Mesa, Arizona, USA) on one adult female in 13 lion prides from 2017 to 2020. Lion locations were recorded at 4-h intervals daily for a total of 60,989 unique locations. Wild dog collars weighed 409 g (< 3% average body weight) and lion collars weighed 740 g (< 1% average body weight). These collars have no detectable effects on wild dog stress hormones, survival, or reproduction and tags of similar relative size have no detectable effects on behavior and space-use of other taxa [12, 49, 75].

We immobilized wild dogs and lions by intramuscular injection of medetomidine and tiletamine—zolazepam, reversing the medetomidine by intramuscular injection of atipamezole after 45 min to one hour. We delivered anesthetics by darting with an air-powered DanInject rifle. All procedures were performed by an experienced and Zambian-registered veterinarian, in collaboration with the Zambia Department of National Parks and Wildlife, with a protocol approved by the MSU IACUC (approval number 2020–123).

### Criteria for data inclusion

For a valid test of the effects of dominant competitors and other variables on wild dog movements, it was important to restrict the analysis to times and places in which all of the variables were well measured. It was not possible to uniformly monitor all carnivore groups and areas in the study tract equally, and we wanted to avoid incorporating data from times and places where less intensive monitoring might be interpreted as a lack of use by lions or wild dogs. To account for yearly variation in monitoring effort and investigate different timescales at which lion use might affect wild dog movements, we restricted our analysis to areas with well monitored groups of both wild dogs and lions, and aggregated data over two time-intervals (one year and six months). We used dBBMMs (see *Dynamic Brownian Bridge Movement Models* below) to create 95% isopleths for each monitored lion pride within each time-interval. We converted these isopleths to polygon shapefiles in QGIS 3.18.3 (www.qgis.org) and combined isopleths that overlapped to delineate a study area within which lion space-use was well described. Wild dog locations that fell within these polygons were included in the analysis. We excluded wild dog locations outside of these polygons unless they fell in well-monitored areas known to have no resident lion prides. For example, a newly formed wild dog pack established a home-range primarily within the Shinganda conservancy where there are no resident lions, as shown by almost a decade of camera trap data from preserve managers and wildlife scout reports.

### Dynamic Brownian bridge movement models

To test how various factors affected wild dog movement in the GKE, we fit dynamic Brownian bridge movement models using the R package *move* [38] to calculate the Brownian motion variance at each location along the movement path of each GPS collared wild dog. Brownian bridge movement models (BBMM) improve upon kernel UD’s by incorporating the sequence of locations and time between them to estimate a constant Brownian motion variance for an animal path [33]. DBBMM’s extend static Brownian bridge movement models by allowing the estimate of Brownian motion variance to vary through time [37]. The estimated Brownian motion variance is affected by the speed and angle of movement [37] and provides a measure of spatial displacement, so that a larger Brownian motion variance implies that an animal is less likely to be close to its prior location. DBBMM’s estimate motion variance by incorporating behavioral change-point analysis [31], which identifies breakpoints in movement patterns along a trajectory. Breakpoints are identified by comparing movements within a specified window of consecutive locations to a specified margin of prior locations directly preceding the window [37]. Simulations and application to real data have shown that dBBMM’s assess space use well, by using information from consecutive locations to assess the likelihood that any given location might have been used in the period between consecutive locations. The long-term utilization of space is well described by dBBMMs, and the motion variance at each location simultaneously provides a simple measure of the magnitude of movement [37].

We fit dBBMMs for both wild dogs and lions from 2017–2020. We then tested for bottom up, competitive, and anthropogenic effects on wild dog movements by fitting a generalized linear mixed effects model (see *Statistical Modelling* below) to the dBBMM motion variance values. Following guidance from Kranstauber et al. [37], we used a biologically relevant timeframe to define the windows and margins used to detect breakpoints in movement patterns within the dBBMM. Increasing window size increases the reliability of motion variance estimates but decreases power to detect small changes in movement patterns [37]. To balance these effects, we selected a margin size of 48 h and window size of one week for both wild dogs and lions, and assessed UD’s visually [37]. These intervals equate to a margin of 5 locations and a window of 15 locations for wild dogs, and a margin of 7 locations and a window of 35 locations for lions. The mean location error for a random subset of 10,000 locations was 1.89 m and the mode was 1 m. We set location error to 1 m when fitting the dBBMM because error associated with resolved quick fix protocol GPS locations is very small relative to movements of wild dogs and lions at this time scale, which are typically hundreds to thousands of meters [65].

### Lion encounter risk

We investigated both reactive and proactive responses [8] of wild dogs to lions. We predicted that wild dogs would proactively respond to areas with a high risk of encountering lions, measured by the local intensity of use from lion dBBMMs. Within the areas that met the criteria for inclusion (described above), we used the raster package in R to sum raster layers of lion use from the dBBMMs fit to locations from each pride [32]. This created a single raster layer of space-use by all lion prides in the study area over a defined time-interval.

Lions use different parts of their home-range at different intensities throughout the year, for example in response to seasonal changes in the distribution of prey [44, 67]. Consequently, we tested whether the time scale over which lion data were aggregated changed the observed response of wild dogs to space use by lions. We created lion space use raster layers from dBBMMS fit to locations over intervals of one year and six months respectively, providing a total of three one-year raster layers and seven six-month raster layers (satellite collars were deployed in the last half of 2017) over the study period. Lion usage values were extracted from the annual and six-month raster layers at every wild dog location in the restricted study area using the raster package in R [32]. This provided two measures of the long-term usage by lions for each wild dog location, allowing us to test if the assessment of risk by wild dogs varied depending on the timescale examined.

We also predicted that wild dog movements would respond reactively to short-term presence of lions by moving quickly to avoid risk once it was detected. To measure reactive responses of wild dogs to lions we calculated the distance in meters from every wild dog location (within the restricted study area) to the closest lion location within a 6-h window. We then classified these distances as near (≤ 2 km) and far (> 2 km). We dichotomized this variable because a six-hour time window provides a rough measure of the true closest approach between wild dogs and lions (at a time other than the known locations). Both species are known to respond to smells or sounds at a distance of two kilometers (or more) [50, 58, 72], and the fact that we did detect a strong response of wild dog movements to this variable (see *Results*) eliminates an otherwise reasonable concern about Type II error due to undetected interactions.

### Prey density, anthropogenic effects and pack composition

We tested the effects on wild dog movement of biotic and abiotic variables previously shown to predict density and distribution of 10 important prey species for wild dogs and lions in Kafue National Park [70]. We also tested for effects of anthropogenic variables, and variables related to pack composition. Values for spatially-explicit variables were extracted from GIS layers at each wild dog location within the study area, and included (1) distance to the park boundary, (2) land-use classification (National Park, Game Management Area, Unprotected), (3) distance to the nearest road, (4) distance to the Kafue River (the largest river in the area), (5) distance to any waterway or tributary, (6) season, (7) breeding status of the pack (breeding pack or single-sex/newly formed group without an established breeding pair), (8) the presence or absence of pups. We calculated distances using the sp [54] & rgeos packages [3] in R. Season was categorized as ‘wet’ for December 1st–May 1st, and ‘dry’ for May 2nd – November 30th. Breeding status of the pack was categorized as ‘breeding’ (groups with a stable alpha pair and established territory) or ‘non-breeding’ (single-sex groups or newly formed packs that had not yet bred and were establishing a territory). Pups were classified as present if the pack had accompanying pups, from the time they left the den to the end of the calendar year, at which point pups were roughly 6-months old and capable of following pack movements.

We divided vegetation into 3 dominant cover types (closed woodland and forest, open woodland, and open grassland), known to influence herbivore density and distribution in the GKE [70]. Vegetation type at each wild dog location was extracted from a raster layer created for the Kavango-Zambezi Transfrontier Conservation area from remote sensed data in 2016 (https://panda.maps.arcgis.com/home/item.html?id=b9459f0149794320b9cf7cc15935e858, accessed June 7, 2021).

### Statistical modelling

**Fig. 1:**
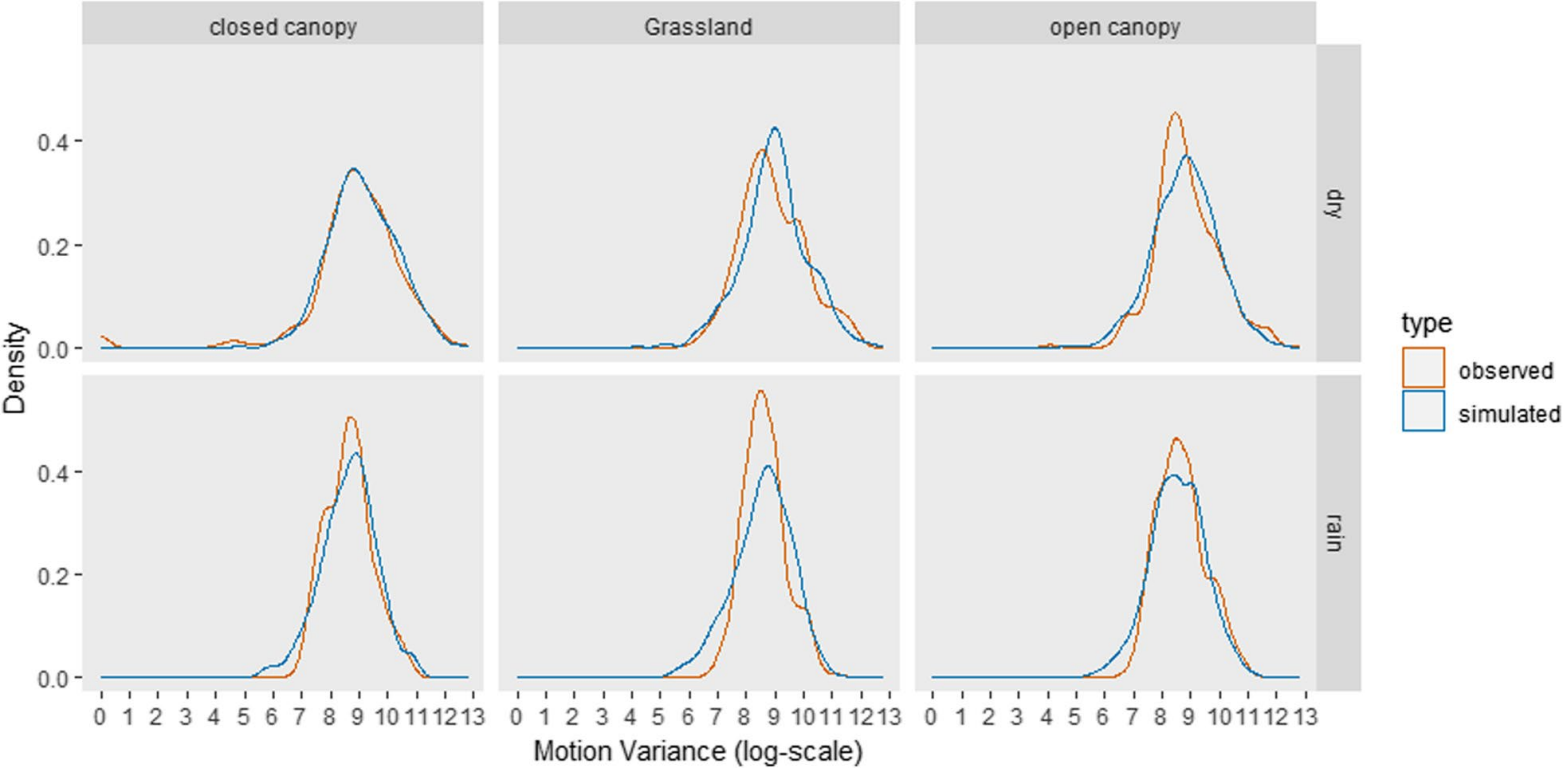
An assessment of the goodness of fit of our full-year negative binomial generalized linear mixed model. For six combinations of season and habitat type, the distribution of y-hat values from the model (orange) closely matched the distribution of observed values (blue)

Using a hypothesis testing approach, we examined a single model with the predictors that we hypothesized could affect wild dog movement. We tested the effects of these variables (described above) on the Brownian motion variance of wild dogs using a negative binomial GLMM (after rounding the Brownian motion variance to integer values) with the default quadratic parameterization (including a random effect of pack identity) fit with the lme4 package v.1.1–27.1 in R version 4.0.2 [2]. Rounding to integers had a trivial effect on the information content of the data, because values ranged from zero into the hundreds of thousands. We compared this model to a negative binomial GLM fit to the original data (i.e., discrete at the scale of measurement) and found no changes in estimated effects, but a slightly worse fit. Finally, we fit a GLM using a gamma distribution. For all models, we assessed model fit by comparing the distributions of values simulated by the model to the original data. We assessed goodness of fit for subsets of the data defined by levels of categorical predictors, using the simulate function of the glmmTMB package [5]. These plots confirmed the negative binomial model (Fig. 1.) fit the data better than the gamma model (Additional file 1: Fig S1.), but the two models produced almost identical estimates of effects on movement (Table 1, Additional file 1: Table S1.). We centered and scaled all continuous predictors before fitting the model, both to improve convergence and to allow direct comparison of the strength of effects on wild dog motion variance. We log transformed the long-term usage of a location by lions because we expected a saturating effect of this variable. We included a random effect of pack identity on the model’s intercept to avoid pseudo-replication, and confirmed that inclusion of this random effect was supported by Akaike’s Information Criterion (AIC) scores. We tested for multicollinearity and found that all generalized variance inflation factor values were less than 5.

**Table 1:** Effects on wild dog Brownian motion variance of variables related to the local risk of lion encounter, prey density and anthropogenic effects

Variable	Estimate (b)	SE	Z-score	*P* value
(Intercept)	9.471	0.139	67.987	< 0.001
**Lion utilization value (log scale)**	**− 0.103**	0.019	**− **5.352	< 0.001
Distance to tributary	0.028	0.012	2.396	0.017
Distance to Kafue river	0.023	0.024	0.975	0.330
Distance to national park boundary	**− **0.013	0.018	**− **0.732	0.464
Distance to road	**− **0.024	0.023	**− **1.059	0.290
Designation: national park	**− **0.119	0.061	**− **1.971	0.049
**Designation: no protection**	**0.431**	0.148	2.923	0.005
**Season: wet**	**− 0.309**	0.025	**− **12.426	< 0.001
Vegetation: grassland	**− **0.0001	0.030	0.004	0.996
Vegetation: open canopy	**− **0.030	0.028	**− **1.049	0.294
**Reproductive status: pups present**	**− 0.355**	0.027	**− **12.977	< 0.001
**Breeding status: non-breeding**	**1.027**	0.042	24.584	< 0.001
**Lion proximity: close**	**0.261**	0.080	**− **3.268	0.001

Coefficient estimates with associated standard errors (SE), Z-scores, and *P* values, for data aggregated over periods of one year. Bold lettering denotes P < 0.01

## Results

### Effects of lions on wild dog movement

**Fig. 2:**
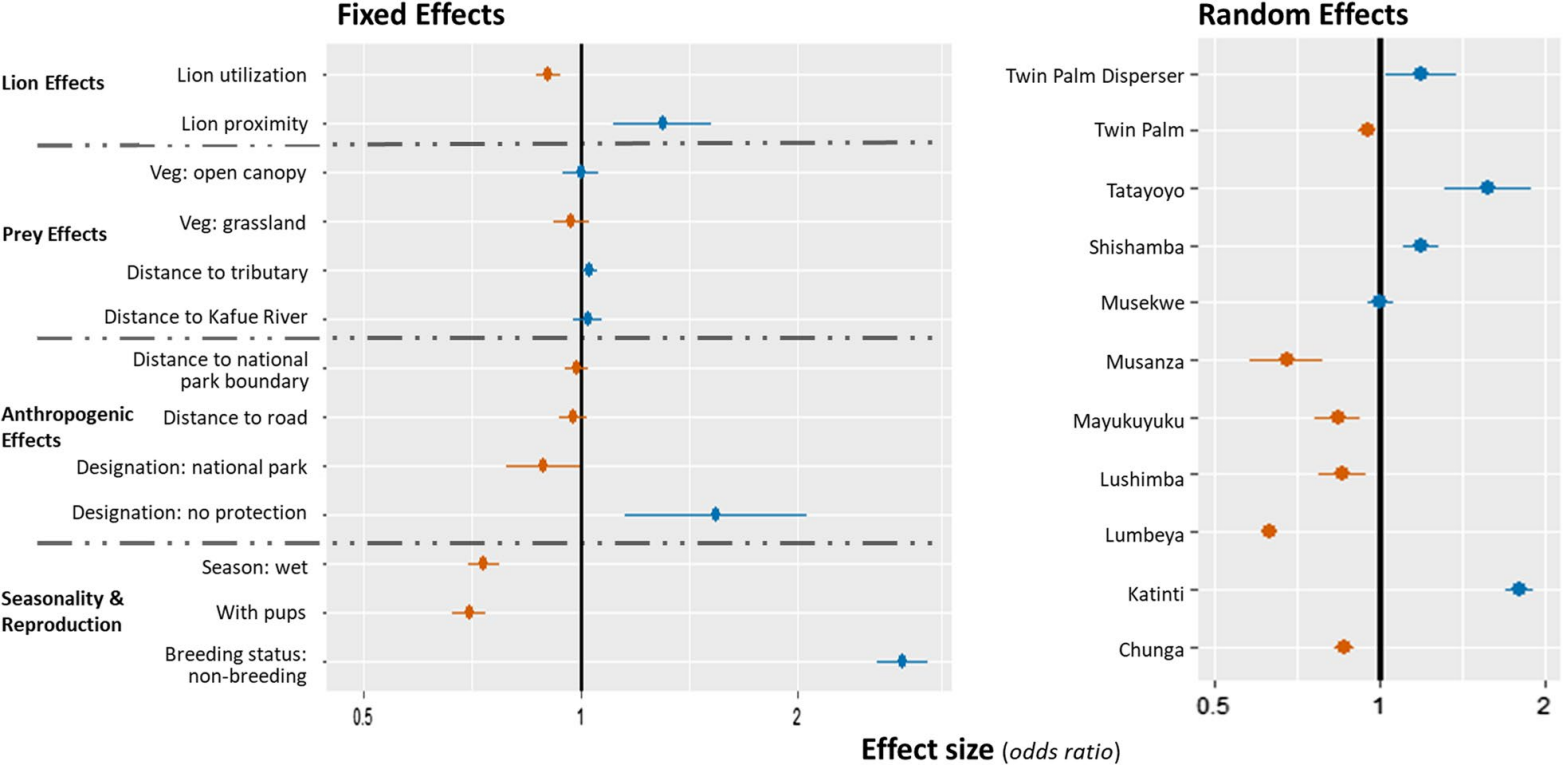
Effects from a generalized linear mixed model (fit to data aggregated over 1-year intervals) of wild dog movements as measured by Brownian motion variance. (Left) Fixed effects, grouped into distinct categories including lion effects (long-term and short-term risk), prey effects (predictors of prey density in Kafue National Park), anthropogenic effects, and effects of seasonality & reproduction (breeding status of pack, and pups present or not). The dark vertical line separates positive and negative parameter effects on motion variance. (Right) Random effects of pack identity (included to avoid pseudoreplication)

Despite the low density of lions in KNP, wild dog movements responded strongly to the presence of lions at both time scales we examined (Tables 1, 2, Fig. 2). Wild dogs showed opposing responses to the short-term risk of encountering nearby lions and the long-term usage of a location by lions, and these responses were very similar when tested with data aggregated over a full year or with data aggregated over six months. Motion variance decreased in areas with a high probability of lion usage over a year (*b* = − 0.10, SE = 0.019 z = − 5.35, *p* < 0.001) or six months (*b* = − 0.12, SE = 0.013, z = − 9.17, *p* < 0.001). Motion variance increased when lions were nearby on the morning or evening that a movement was made, and (as expected) this result was similar for models fit to data from 12-month periods (*b* = 0.26, SE = 0.080, z = − 3.27, *p* = 0.001) or six-month periods (*b* = 0.25, SE = 0.087 z = − 2.87, *p* = 0.004).

**Table 2:** Effects on wild dog Brownian motion variance of variables related to the local risk of lion encounter, prey density and anthropogenic effects

Variable	Estimate	SE	Z-score	*P* value
(Intercept)	9.528	0.177	54.154	< 0.001
**Lion utilization value (log scale)**	**− 0.115**	0.013	**− **9.166	< 0.001
Distance to tributary	**− **0.002	0.012	**− **0.355	0.889
**Distance to Kafue river**	**0.087**	0.025	3.493	< 0.001
Distance to national park boundary	**− **0.045	0.019	**− **2.375	0.013
Distance to road	0.036	0.019	1.874	0.061
Designation: national park	**− **0.094	0.093	**− **1.004	0.316
Designation: no protection	**− **0.089	0.200	**− **0.446	0.656
**Season: wet**	**− 0.411**	0.025	**− **16.358	< 0.001
Vegetation: grassland	**− **0.005	0.032	0.163	0.871
Vegetation: open canopy	**− **0.058	0.030	**− **1.918	0.055
**Reproductive status: pups**	**− 0.215**	0.030	**− **7.144	< 0.001
**Breeding status: non-breeding**	**0.864**	0.045	19.253	< 0.001
**Lion proximity: close**	**0.251**	0.087	**− **2.872	0.004

Coefficient estimates with associated standard errors (SE), Z-scores, and *P* values, for data aggregated over periods of 6 months. Bold lettering denotes *P* < 0.01

### Effects of variables known to predict prey density on wild dog movement

Wild dog movements showed inconsistent and weak responses to a set of ecological variables previously shown to predict the local densities of their primary prey. This result was consistent for models fit to data aggregated over a year or a six-month season. Motion variance did not detectably differ among vegetation classes (Table 1, 2). Motion variance was affected by the distance to the nearest river or any tributary, but this effect was not consistent across time scales (12-month analysis: *b* = 0.028, SE = 0.012 z = 2.396, *p* = 0.017; 6-month analysis: *b* = − 0.002, SE = 0.012, z = − 0.355, *p* = 0.889). Wild dog movements increased when they were far from the Kafue River when tested with data aggregated over a season (6-month interval) (*b* = 0.087, SE = 0.025, z = 3.49, *p* < 0.001) but this effect was considerably weaker when tested with data aggregated over a year (*b* = 0.023, SE = 0.024, z = 0.975, *p* = 0.330). Overall, variables that predict local prey density had much weaker effects than variables that predict the risk of encountering lions.

### Anthropogenic effects on wild dog movement

Wild dog movements changed weakly when they moved between the (strictly-protected) National Park, (multiple-use) Game Management Areas, and unprotected areas over 6-month timescales (Tables 1, 2). At the12 month timescale, motion variance increased in unprotected areas, relative to the NP or GMAs (*b* = 0.43, SE = 0.148, z = 2.92, *p* = 0.005). For data aggregated over a year, neither distance to the nearest road nor distance to the park boundary (when inside the NP) had detectable effects on wild dog motion variance (Table 1). For data aggregated over six months, motion variance increased when wild dogs were far from roads (*b* = 0.036, SE = 0.019, z = 1.87, *p* = 0.061) and decreased as they approached the park boundary (*b* = − 0.045, SE = 0.019, z = − 2.38, *p* = 0.013), but both of these effects were relatively weak.

### Effects of group structure and reproductive state on wild dog movement

The distinction between single-sex groups and established breeding packs had the largest effect on motion variance of any variable we examined, at both 12-month (*b* = 1.03, SE = 0.042 z = 24.59, *p* < 0.001) and 6-month (*b* = 0.86, SE = 0.045, z = 19.25, *p* < 0.001) timescales. Single-sex groups of dispersers had much larger motion variances than established breeding packs. Within established packs, motion variance decreased substantially when they had accompanying young pups at both the 12-month (*b* = − 0.36, SE = 0.027 z = − 12.98, *p* < 0.001) and 6-month timescale (*b* = − 0.22, SE = 0.030, z = − 7.14, *p* < 0.001). Season had the second strongest effect on motion variance at both timescales, revealing a substantial decrease in movement during the rainy season (12 month: *b* = − 0.31, SE = 0.025, z = − 12.43, *p* < 0.001) (6 month: *b* = − 0.41, SE = 0.025, z = − 16.36, *p* < 0.001).

## Discussion

**Fig. 3:**
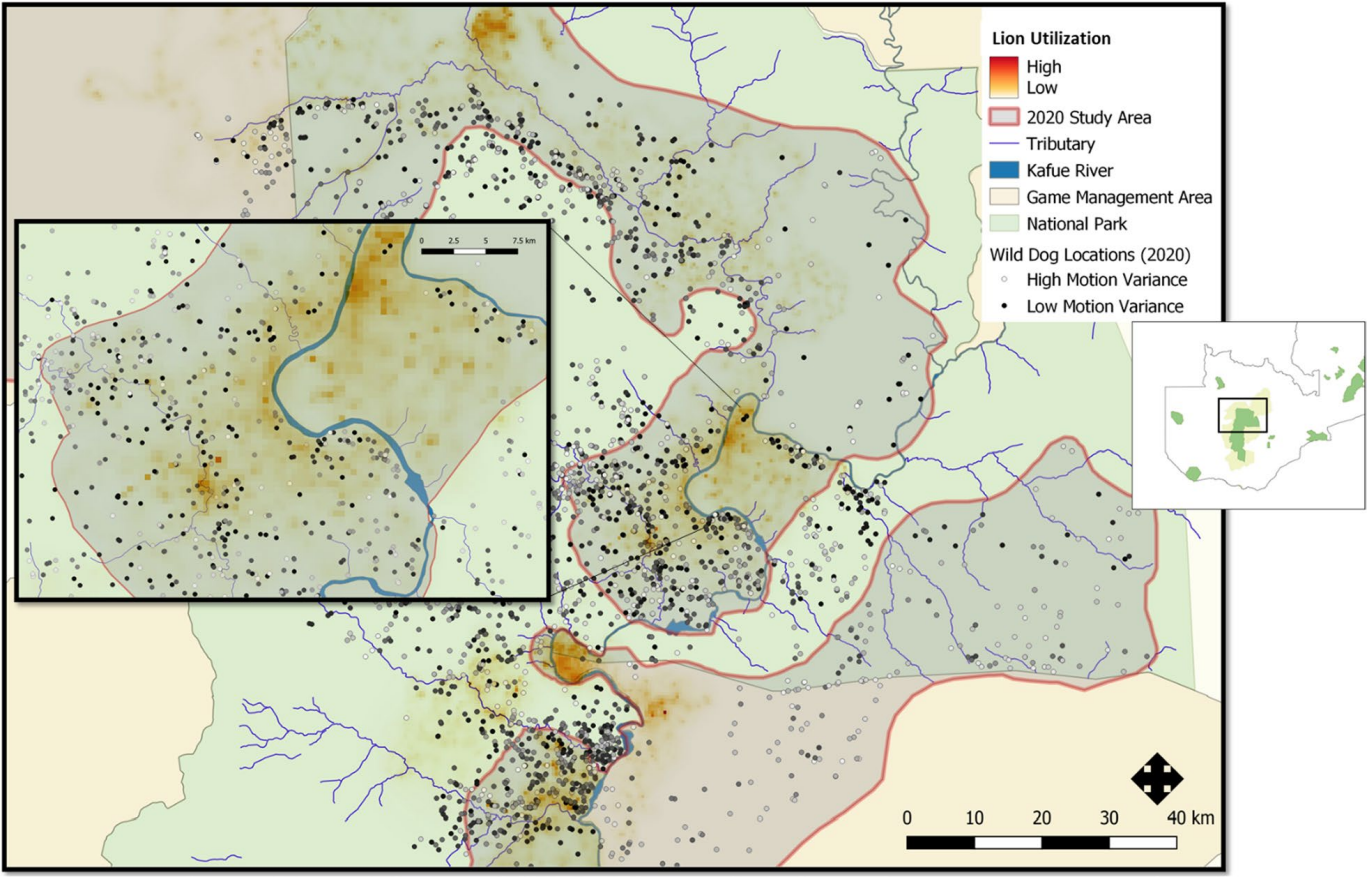
2020 Wild dog locations, lion utilization from a dBBMM, and the study area boundaries. Wild dog locations (points) are color-coded to show the dynamic Brownian motion variance at each point. Lion utilization values (background shading) show the long term use of each pixel derived from dBBMM. Study area is delineated as transparent grey with red borders (see criteria for data inclusion). The inset map at right show the location of the main map within Zambia, with National Parks shown in green and the Game Management Areas that border Kafue National Park shown in yellow. The inset map at left magnifies a central portion of the study area and shows (a) that wild dogs tend to avoid areas that are highly used by lions (relatively few points fall within heavily shaded areas) and (b) that wild dogs’ Brownian motion variance was low when they were in areas that are highly used by lions (wild dog points are darker in heavily shaded areas)

Wild dogs altered their movements in response to the long-term usage of an area by lions, and in response to the immediate proximity of lions. Recall that a reduction in motion variance implies that an animal shows less spatial displacement between consecutive locations. Wild dogs proactively decreased motion variance in areas of high lion density (high long-term risk of encounter) (Fig. 3). Wild dogs reactively increased motion variance when they were close to lions in both space and time (high short-term risk of encounter). Thus, wild dogs showed contrasting proactive and reactive responses to long-term and short-term variation in risk, a result that was consistent for analyses over 6-month and 1-year intervals. Both of these responses could affect home range size, spatiotemporal overlap with competitors, and access to prey (because dominant competitors typically select areas with high resource availability). Given wild dogs’ limited capacity to compensate for problems that exacerbate an already tenuous energy budget [11, 29], the observed increase in motion variance in response to short-term risk could carry meaningful energetic costs, independent of any effect on spatial distributions. The observed decrease in motion variance when moving through areas that were heavily used by lions would reduce the rate of energy expenditure, but might also reduce rates of encounter with prey. These possibilities warrant further investigation.

On this study site in the GKE, the densities of wild dogs’ preferred prey species are predicted by habitat type and proximity to water [70]. Habitat type had no detectable effect on wild dog motion variance at either timescale in this analysis, but motion variance did show a positive relationship with distance to the Kafue River (i.e., wild dogs slowed down and had more directed movements when close to the largest river in the national park). We detected this effect only at the 6-month timescale and not at the annual time scale. These differing results could arise because the 6-month timescale better captures seasonal changes as prey (and lions) make seasonal movements that are tied to reliable permanent water sources [16, 66]. The GKE is characterized by a pronounced wet season in which herbivores are widely distributed across the landscape, and a dry season in which herbivores concentrate around water. Overall, the results for proximity to water suggest that the effects of water on prey distributions might affect wild dog movements (as would be expected), but these effects are weaker and more variable than the effects of lions. This possibility also warrants further investigation.

**Fig. 4:**
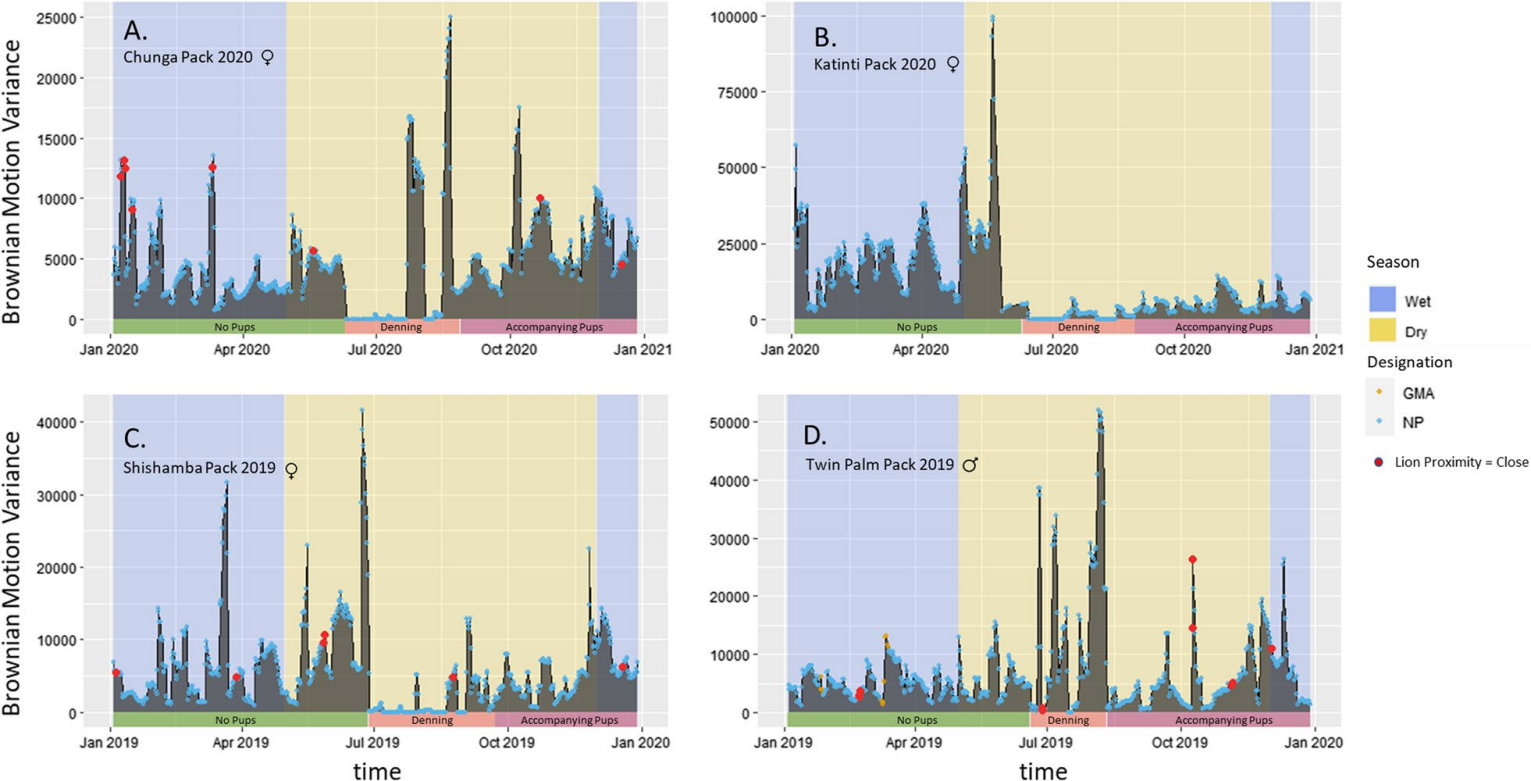
Changes over time in wild dog movements (as measured by Brownian motion variance) for specific packs, each over one year. Motion variance is plotted on the ordinate as it changes over time. Background shading of the plot frame shows the wet (blue) and dry (yellow) season. The colors of the bar at the bottom show the reproductive status of the pack (no accompanying pups (green), denning (orange), and with accompanying pups (purple). In the packs A, B & C, the alpha female was radio-collared; in pack D, the alpha male was collared. See methods for the details of analysis that addressed this difference. Large red dots denote cases in which a pack was known to be within 2 km of a monitored lion pride. Color of the points excluding red dots indicate designated protection status of the area (blue: within the National Park, orange: within Game Management Areas)

Covariates related to group structure, reproductive state, and seasonality all had strong effects on wild dog motion variance (Fig. 4). Wild dogs in the GKE reduced movement during the wet season (Tables 1, 2). We suggest two possible explanations for this result. First, movement can be difficult in the wet season due to extensive flooded regions and the prevalence of thick grasses up to 3 m in height which would increase energetic costs. Second, movement through tall grass impedes the ability of wild dogs to scan for risks (and prey) and increases the risk of predation from stalking predators such as lions [25]. Thus, it is possible that decreasing movement during the wet season is a mechanism to conserve energy and reduce predation risk.

As expected, non-resident and dispersing groups of wild dogs had markedly larger motion variance than resident breeding packs, similar to prior studies investigating dispersal [7, 76]. In the GKE, this distinction had the largest effect on movement of any variable we examined. Prior studies have also shown changes in movement and habitat selection by wild dogs when they are denning or accompanied by small pups just after denning [10, 24, 34, 55]. When breeding packs in Kafue had accompanying pups, motion variance was significantly reduced, almost certainly so that pups could keep up with the pack, and perhaps also as a proactive response to risk when the wild dogs are at their most vulnerable phase. For example, wild dogs in Kruger National Park tolerated less risk by reducing site revisitation rates during denning periods [45]. Pup mortality generally increases as a result of predation from lions after the denning period,when pups are small, slow, and inexperienced, but without the protection of a den [30]. The observed reduction of motion variance for packs with accompanying pups could be a proactive response to risk (similar to their response to areas with high lion density), but in this case reflecting increased sensitivity to risk due to the high vulnerability of pups. Although periods of “accompanying pups” primarily occurred in the dry season, packs without pups showed an increase in motion variance during the dry season, while packs with pups showed a reduction in motion variance when the rains began, especially when compared to pre-denning movement rates (Fig. 4). By using data from both non-breeding and breeding groups in both seasons, we were able to resolve the marginal effects of season and reproductive state.

Anthropogenic variables such as roads and the national park boundary had little effect on the motion variance of wild dogs in the GKE, and only at the 6-month timescale. Wild dog motion variance increased as the level of protection decreased and was substantially higher in non-protected areas than the national park and associated GMAs. While motion variance did not differ detectably between national parks and GMA’s in this analysis, we suggest caution when interpreting this result. The increase in motion variance in GMAs vs the national park was comparable to the effect size of other variables. Additionally, substantial data from the GMAs came from well-protected areas, which in some cases had better protection than certain portions of the national park. Heavily-impacted GMAs rarely support wild dogs, and when they do, observed motion variances would likely show patterns similar to what we found in unprotected areas. These three designations were the most suitable aggregations of the available data, but it is possible that upon further investigation, effects on motion variance between National Parks and GMAs could be altered. Hidden Markov models showed similar results for wild dogs in the Luangwa Valley, with increased movement speed in GMAs relative to the better-protected South Luangwa National Park, probably because prey density was lower and because dispersing groups often travelled through Game Management Areas [14, 59]. Similarly, dispersing groups in our study often made forays into GMAs and non-protected areas in search of new territories, however resident packs did use GMA’s extensively as well. Creel et al. [14] also found that wild dogs decreased their speed of movement when entering areas with high human footprint index (HFI) values, similar to the response of wild dogs to areas that were heavily used by lions in this study. Wild dogs in this study did not enter areas with high HFI values often enough to test its effect on their movements.

Prey depletion in Kafue National Park and surrounding Game Management Areas has reduced prey populations and altered prey community structure [13, 70]. This reduction of prey has had substantial negative effects on the large carnivore community, lion density is 3.4 times lower than comparable ecosystems, and wild dog density is 4.8 times lower [28, 69]. The GKE’s wild dog population is characterized by large home-ranges with minimal overlap, small pack sizes, and survival rates comparable to systems with higher wild dog density [28]. Together with the effects of lions on wild dog movement patterns described here, these studies suggest that the effect of interspecific competition with lions on the wild dog population of the GKE likely remains strong, even though lion numbers have been greatly reduced by prey depletion. Although lion density in the GKE is approximately one-third of their density in comparable ecosystems, wild dogs still moved slowly in areas with high risk of encountering lions, and moved quickly when lions were immediately close.

It is likely that lions can detect spatiotemporal variation in prey density more accurately than the predictors of prey density in our model. Because lions preferentially select areas with high prey density, wild dogs are known to encounter more prey in areas with high lion density [26]. Consequently, it is possible that some portion of the reduction in wild dog movement in risky areas is due to higher-than-predicted prey density in areas that are preferentially used by lions. While it is clear that risk from lions has strong effects on wild dog movements after controlling for variables known to affect prey density, data from areas of high and low use by lions on wild dogs’ hunting effort (distance travelled), fine-scale movement patterns, prey encounter rates, and hunting success would be of value to better understand this effect.

The observation that lions affect the spatial distribution of wild dogs has consistently been reported in many ecosystems [9, 19, 29], but (to our knowledge) this study is the first to test how lions affect their movements (Fig. 4). Very few studies of any species have examined changes of movement in response to both short- and long-term variation in risk, but limited data reveal that several species proactively respond to risk in a manner similar to wild dogs. Wildebeest (*Connochaetes taurinus*) showed responses to predators that were similar to wild dogs’ responses to dominant competitors: they slowed down in areas of high long-term risk and speeded up in response to high short-term risk [18, 19]. King cobras (*Ophiophagus hannah*) showed a reduction in motion variance in human dominated agricultural areas [46]. Mountain lions (*Puma concolor*) decreased movement rates when in close proximity to cues of human use [62], but increased movements rates dramatically in response to a direct encounter with human hunters (Williams et al. 2020). Finally, increased movement rates are thought to increase the mortality rates of bobcats (*Lynx rufus*) by increasing exposure to human-caused mortality [6].

We suggest that decreasing motion variance when moving through an area with high long-term risk could arise from cautious movement that allow better detection and assessment of risk when moving from one location to another. We suggest that increased motion variance in response to an immediate nearby risk, probably arises from fleeing or retreating from a threat that has been detected and assessed [18]. Some other competitively subordinate carnivores, particularly felids like the cheetah and leopard, avoid dominant competitors through immediate, short-term adjustments to space use [4, 19, 68]. Social canids, such as wild dogs, are behaviorally less cryptic and thus less likely to rely on immediate, short-term avoidance. Instead, we demonstrate here that wild dogs respond to temporal and spatial proximity of lions with substantial increases in movement. This confirms direct observations of cases in which wild dogs detect lions (or experimental playbacks of lion roars) nearby and respond by reversing their direction and moving quickly for a large distance [10, 72]. To avoid the risk of direct predation or energetically costly rapid flight, wild dogs may benefit by proactively reducing motion variance to allow better risk assessment when moving in high-risk areas. Such effects on movement may indicate that competitive limitation of wild dogs by lions remains strong in prey depleted systems, even though lion density is significantly reduced.

## Conclusion

Movement analyses can provide valuable insights into the behavioral responses of a species to the spatial distribution of benefits (e.g., prey) and costs (e.g., competitors or predators) [73]. Dynamic Brownian Bridge movement models have proven effective for home range estimation in a wide range of species [37, 60], and have been effective in identifying hotspots, corridors, and avoidance/attraction behavior [39, 52, 71]. The Brownian motion variance calculated along an individual’s movement path also provides valuable information that can be tested against a wide range of covariates [37]. While we have focused on comparing the effects of competitors, prey, and humans, such tests can be applied to almost any animal to give valuable insight into the variables that alter movement, with consequences for habitat selection, space use, species interactions, and landscape connectivity.

We found that that wild dog movements in a prey-depleted system remain heavily influenced by lions, even though lion density is three times lower than ecologically comparable ecosystems. Subtle costs of competition with lions may be two-fold: 1. High energy expenditure during large, fast movements in reaction to close proximity to lions, 2. Sub-optimal hunting as a consequence of proactively reduced movement in lion dense areas. These results have immediate conservation implications because wild dogs and many subordinate carnivores, are increasingly affected by prey depletion across their range [74]. It has been well established that the elimination of a dominant competitor can release subordinates, with cascading effects on other species [57]. However, if the reduction of dominant competitor densities is caused by prey depletion, it does not necessarily allow competitive release [28]. Even at low densities, costs imposed on wild dogs by their dominant competitors appear to remain strong, and may partially explain one of the mechanisms that inhibit competitive release of wild dogs in prey depleted systems.

## Supplementary Information

**Additional file 1.** **Figure S1**. An assessment of the goodness of fit of our full-year generalized linear mixed model using a gamma distribution. For six combinations of season and habitat type, the distribution of y-hat values from the model (orange) does not match the distribution of observed values (blue) as well as the negative binomial model. **Table S1**. Effects on wild dog Brownian motion variance of variables related to the local risk of lion encounter, prey density and anthropogenic effects using a model fit with a gamma distribution. Coefficient estimates with associated standard errors (SE), Z-scores, and P-values, for data aggregated over periods of one year are consistent with results of the negative binomial model. Bold lettering denotes P < 0.01.

## Data Availability

All data analyzed during the current study are available upon reasonable request.
