# Supplementary material for: African wild dog movements show contrasting responses to long and short term risk of encountering lions: analysis using dynamic Brownian bridge movement models
Source: Mov Ecol. 2022 Mar 31;10:16. doi: 10.1186/s40462-022-00316-7 (PMC8974231; doi:10.1186/s40462-022-00316-7)
Supplement: Supplementary file 1 — Additional file 1. Figure S1. An assessment of the goodness of fit of our full-year generalized linear mixed model using a gamma distribution. For six combinations of season and habitat type, the distribution of y-hat values from the model (orange) does not match the distribution of observed values (blue) as well as the negative binomial model. Table S1. Effects on wild dog Brownian motion variance of variables related to the local risk of lion encounter, prey density and anthropogenic effects using a model fit with a gamma distribution. Coefficient estimates with associated standard errors (SE), Z-scores, and P-values, for data aggregated over periods of one year are consistent with results of the negative binomial model. Bold lettering denotes P < 0.01. [file 40462_2022_316_MOESM1_ESM.docx]

**Supplementary Material**

**
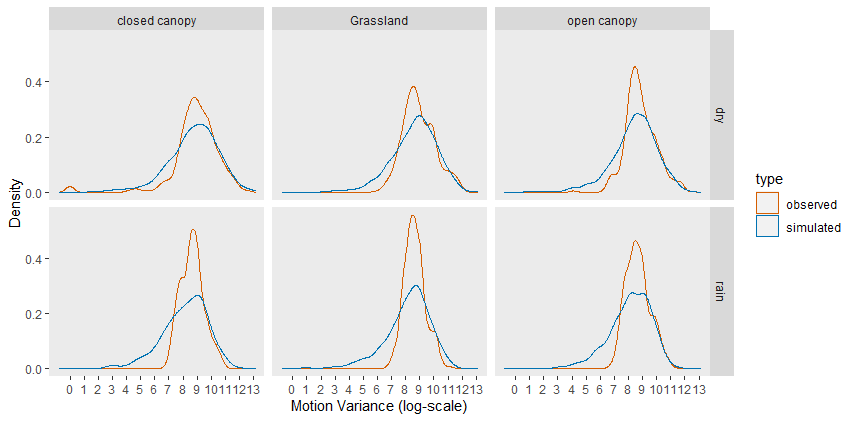
**

Figure S1. An assessment of the goodness of fit of our full-year generalized linear mixed model using a gamma distribution. For six combinations of season and habitat type, the distribution of y-hat values from the model (orange) does not match the distribution of observed values (blue) as well as the negative binomial model.

| Variable | Estimate (b) | SE | Z-score | P-value |
| --- | --- | --- | --- | --- |
| (Intercept) | 9.471 | 0.141 | 66.871 | < 0.001 |
| **Lion utilization value (log scale)** | **-0.103** | 0.019 | -5.353 | < 0.001 |
| Distance to tributary | 0.028 | 0.012 | 2.407 | 0.016 |
| Distance to Kafue river | 0.023 | 0.024 | 0.967 | 0.333 |
| Distance to national park boundary | -0.013 | 0.018 | -0.733 | 0.463 |
| Distance to road | -0.024 | 0.023 | -1.080 | 0.280 |
| Designation: national park | -0.120 | 0.060 | -1.979 | 0.048 |
| **Designation: no protection** | **0.430** | 0.148 | 2.915 | 0.005 |
| **Season: wet** | **-0.309** | 0.025 | -12.448 | < 0.001 |
| Vegetation: grassland | -0.0001 | 0.030 | 0.000 | 0.999 |
| Vegetation: open canopy | -0.030 | 0.028 | -1.057 | 0.291 |
| **Reproductive status: pups present** | **-0.355** | 0.027 | -12.995 | < 0.001 |
| **Breeding status: non-breeding** | **1.027** | 0.042 | 24.628 | < 0.001 |
| **Lion proximity: close** | **0.261** | 0.080 | -3.265 | 0.001 |

*Table S1. Effects on wild dog Brownian motion variance of variables related to the local risk of lion encounter, prey density and anthropogenic effects using a model fit with a gamma distribution. Coefficient estimates with associated standard errors (SE), Z-scores, and P-values, for data aggregated over periods of one year are consistent with results of the negative binomial model. Bold lettering denotes P < 0.01.*
